# Supplementary material for: Tead1a Initiates Transcriptional Priming Through the TEAD1a/YAP‐Notch1‐Spi1/Cebpα Axis to Promote Neutrophil Fate
Source: Adv Sci (Weinh). 2025 Aug 28;12(41):e05441. doi: 10.1002/advs.202505441 (PMC12591127; doi:10.1002/advs.202505441)
Supplement: Supplementary file 4 — Supplementary Dataset [file ADVS-12-e05441-s004.zip › 3-3.zip!/3-3/02-3.cluster/cluster6/cluster6_featureplot.pdf]

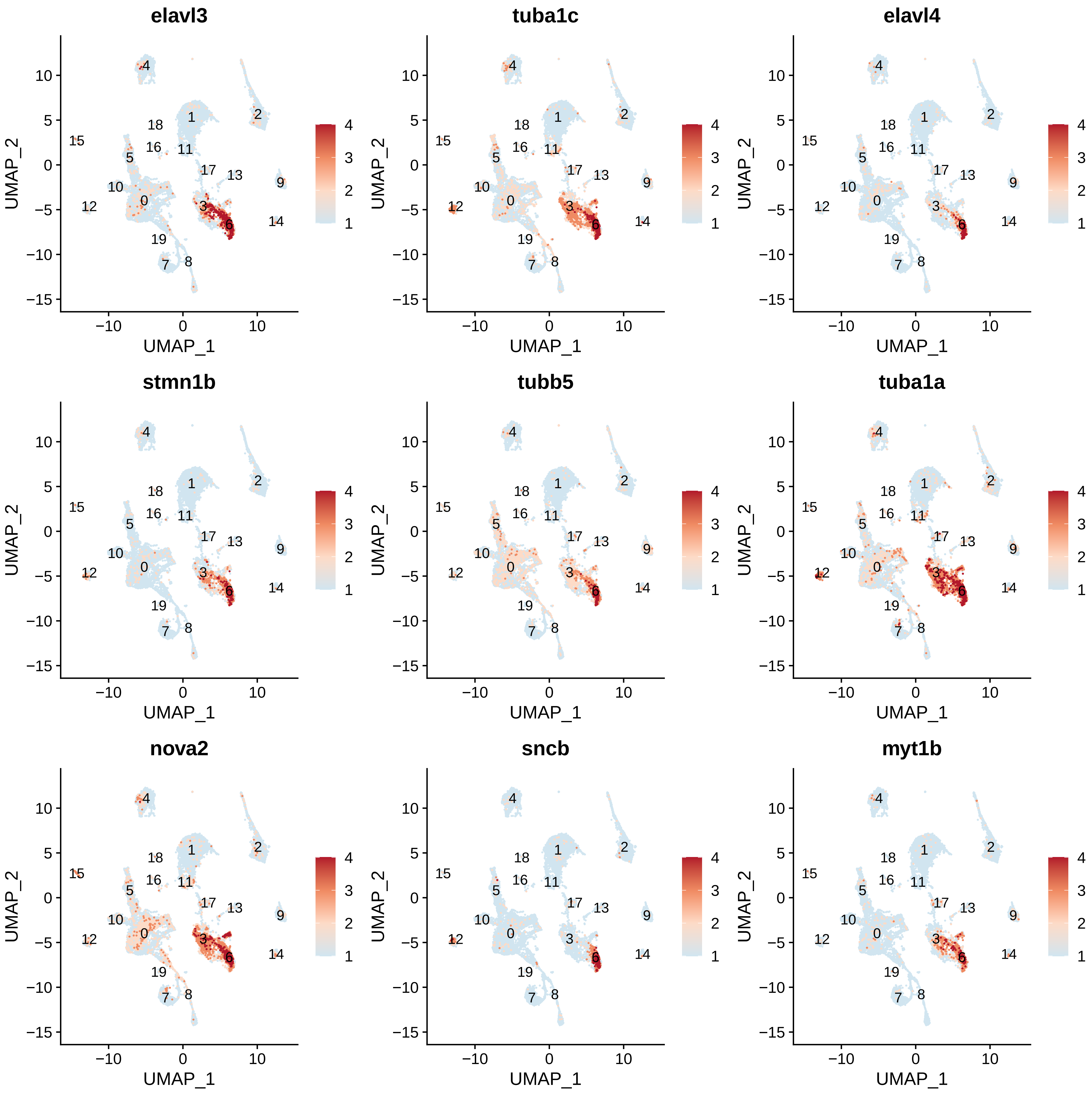
elavl3
tuba1c
elavl4
stmn1b
tubb5
tuba1a
nova2
sncb
myt1b
UMAP_1
UMAP_2
